# Supplementary material for: Identifying researcher characteristics driving growth in Japanese University-originated deep-tech startups: A machine learning approach
Source: PLoS One. 2026 Apr 3;21(4):e0346137. doi: 10.1371/journal.pone.0346137 (PMC13048777; doi:10.1371/journal.pone.0346137)
Supplement: Attachment — Submitted filename: Response_to_Reviewer#2_Mizuhara.docx [file pone.0346137.s002.docx]

# Response to Reviewer 2

We sincerely thank Reviewer #2 for their thoughtful comments and constructive suggestions, which have greatly helped us to improve the clarity and rigor of our manuscript. Below, we provide detailed responses to each point raised. Reviewer comments are shown in *italic*, followed by our responses.

## Comment 1

*“The data sources and data selection are not sufficiently described. There might be selection effects. At least, the authors do not mention any counteractivities (to selection effects) and also do not sufficiently justify and explain their decisions on the selection. From 967 companies, 357 are linked to their DB and only 194 are finally analyzed. This is a huge filter that was applied here. Even within these companies, further selections on the researchers to be analyzed were made. What does this imply?”*

**Response:**
We appreciate the reviewer’s thoughtful comment on our data sources and selection process. We agree that the manuscript did not provide sufficient detail on how the final dataset was constructed, even though the filtering steps themselves were based on methodological necessity to ensure consistency and reliability.
In the revised manuscript, we have clarified the rationale for each filtering stage in Section 3.3 “Data Cleaning” and added explanatory text at Line 169 and Line 174 as follows:

- Line 169: “This reduction occurred because only those startups that explicitly disclosed their researchers in the original database and could be reliably matched to financial records were retained to ensure data consistency and to avoid missing values in explanatory variables.”
- Line 174: “This year corresponds to a major institutional and financial shift initiated by the enforcement of the Industrial Competitiveness Enhancement Act (April 1, 2014) and the launch of the Public-Private Innovation Program by the Ministry of Education, Culture, Sports, Science and Technology (MEXT). Under this program, four major national universities—the University of Tokyo, Kyoto University, Osaka University, and Tohoku University—each established university-affiliated venture capital funds with a total investment capacity of approximately 100 billion JPY, beginning in FY 2014. Therefore, the post-2015 subset represents startups founded under this new policy and funding environment, ensuring analytical consistency within a unified institutional context (21).”

Furthermore, to make the selection process more transparent, we have revised Figure 1 to explicitly indicate the number of startups remaining at each stage (967 → 357 → 194). This visual update helps readers clearly trace the flow of data selection and understand how the analytical sample was constructed.
Through these clarifications, we aim to make it explicit that the filtering steps were methodologically justified and were designed to improve the internal validity of the analysis, rather than reflecting any selection bias.

## Comment 2

*“Furthermore, no counter-factual or control-group approach was implemented, which leaves the reader unsure about the solidity of the findings.”*

**Response:**
We appreciate the reviewer’s helpful comment. We agree that a counter-factual or control-group approach would strengthen the causal robustness of the findings. However, as the present study focuses on identifying researcher characteristics under Japan’s limited data availability, such an approach was not feasible at this stage.
To partly address this limitation, we applied 5-fold cross-validation and compared multiple model settings (Cases 1–4) with consistent results, ensuring internal robustness. We have also clarified in the revised manuscript (Section 6, Conclusion) that the establishment of a counter-factual or longitudinal framework is an important direction for future studies, once more comprehensive national data become available.

## Comment 3

*“In addition, the work is only scarcely embedded in the conceptual literature. For example, work by Lerner (2002) or Mazzucato (2013) on the role of public research for R&D and innovation or by Lockett et al. (2005), Meyer (2003), or Visintin and Pittino (2014) on academic spin-offs is not cited.”*

**Response:**
We sincerely appreciate the reviewer’s valuable comment and the suggested references, which have greatly improved the conceptual foundation and logical structure of the manuscript. In response, we have incorporated the cited works by Lerner (2002), Mazzucato (2013), Lockett et al. (2005), Meyer (2003), and Visintin and Pittino (2014) into the **Introduction** section to more clearly position this study within the broader literature on academic entrepreneurship and public R&D.
Specifically, the following sentences have been added and revised:

- **Lines 13–18:**　“As Lerner (2) emphasized, innovation is driven not merely by institutional reforms but by effective mechanisms that connect academic research, venture capital, and entrepreneurship, highlighting the pivotal role of researchers in transforming scientific knowledge into economic value. Likewise, Visintin and Pittino (3) demonstrated that university-based spin-offs with founding teams combining academic and managerial expertise outperform those composed solely of academics, underscoring that researchers’ active participation is critical for early-stage growth.”
- **Lines 30–40:** “Lockett et al. (10) conceptualized such university-based spin-offs within a knowledge-based view, defining them as independent firms created to commercialize publicly funded research and emphasizing that successful commercialization depends on bridging ‘knowledge gaps’ between scientific and entrepreneurial capabilities. Meyer (11) further highlighted that researchers play diverse roles in the commercialization of academic knowledge, distinguishing between ‘academic entrepreneurs’ and ‘entrepreneurial academics.’ He argued that public support mechanisms can shape these behaviors, emphasizing that managerial expertise and external networks are crucial for successful university spin-offs. … As Mazzucato (16) argued, because the state itself can act as a primary risk-taking agent in innovation, this approach is particularly relevant for Japan.”

Through these additions, the revised manuscript now explicitly situates the study within the conceptual framework of academic entrepreneurship, public R&D policy, and the commercialization of university research.

## Detailed Question 1

*“Line 50: “Therefore, in this study we utilize Japan-specific data to analyze the relationship between deep-tech startup growth and the characteristics of involved researchers,…”. In the Japanese sys-tem, is there a difference to be expected between university spin-offs and other deep tech spin offs?”*

**Response:**
Thank you for pointing out the possible ambiguity. We do not assume systematic differences between university spin-offs and other deep-tech spin-offs in Japan. Our focus on university-originated startups is purely pragmatic: nationally standardized, government-maintained datasets are available only for university-originated ventures, which makes them the sole reliable, comprehensive source for Japan-wide analysis at present.
To clarify this point, we have revised the Purpose section to explicitly state that our study’s scope is determined by data availability and standardization, not by conceptual distinction. The following text has been added in the revised manuscript.

- **Lines 68–71:** “We focus on university-originated startups not because we assume systematic differences from other Japanese deep-tech startups, but because reliable, government-maintained datasets exist only for the university-originated subset, making it the sole nationally standardized source for analysis in Japan.”

We believe this clarification resolves the ambiguity and accurately reflects the rationale for our data selection.

## Detailed Question 2

*“Line 131: “The “University-Originated Venture Database” contained 967 listings, of which 357 startups linked to “STARTUP DB” by corporate number or other identifier had complete researcher data.” Did you conduct an analysis on the missing. Are these systematically biased in any of the relevant dimensions that can still be controlled without the STARTUP DB link?”*

**Response:**
Thank you for the comment. As noted in **Response to Comment 1,** the reduction was due to data reliability requirements, not selective exclusion.

## Detailed Question 3

*“Line 131: “The “University-Originated Venture Database” contained 967 listings, of which 357 startups linked to “STARTUP DB” by corporate number or other identifier had complete researcher data.” Did you conduct an analysis on the missing. Are these systematically biased in any of the relevant dimensions that can still be controlled without the STARTUP DB link?”*

**Response:**
Thank you for this important question. The initial dataset contained 967 entities that were potentially university-affiliated startups. Among them, only 357 could be reliably linked to STARTUP DB, which provides validated information on registered startup firms in Japan. The remaining 610 entities could not be linked because many were not startups in the strict sense—they included small or local companies, university joint ventures, or general incorporated associations without clear startup status. Moreover, publicly available data for these unlinked entities were extremely limited and inconsistent. Therefore, we focused our analysis on the 357 companies that could be verified as startups in STARTUP DB to ensure data reliability and consistency with the study’s focus on university-originated deep-tech startups. This selection reflects the scope of the research rather than an omission of relevant startup data.

## Detailed Question 4

*“Line 134: “… startups founded from 2015 onward …“. Why another reduction of the number of startups and not use the pre-2015 start-ups as a control group?”*

**Response:**
Thank you for the comment. As noted in **Response to Comment 1**, the reduction was due to data reliability requirements, not selective exclusion.

## Detailed Question 5

*“Line 137: “Although some university-originated startups have multiple involved researchers, each researcher was linked to only one startup.” This sentence is unclear. Were all researchers assigned, so when more than one was involved all of them have been included in the analysis?*

*Line 141: “For Positive startups, the researcher with the highest normalized sum was selected, while for Negative startups, the researcher with the lowest sum was chosen. This approach was taken due to the reliance on self-reported data in the “University-Originated Venture Database,” which makes it challenging to objectively identify the most relevant researcher when multiple indi-viduals are involved.” Doesn’t‘ this lead to a tautology?”*

**Response:**
We appreciate the reviewer’s insightful comment regarding potential bias arising from our previous rule for selecting researchers. We agree that choosing the researcher with the highest or lowest normalized sum could lead to data leakage, as it presupposes knowledge of the target outcome.

To address this issue, we revised our data selection process as follows. For startups with multiple researchers, one researcher was randomly selected for model training, ensuring that the selection did not depend on the startup’s outcome label. This random selection was repeated five times, and the average of the model performance metrics (Accuracy, Recall, Precision, F1, AUC) across the five iterations was reported.

The results showed no substantial change in accuracy, but we observed a trend of higher Recall and slightly lower Precision, indicating a reduction of overfitting. The revised results are reflected in the new Table 4, and related Figure 2–6.

Importantly, the interpretation and discussion in Section 5 (“Consideration of Researcher Characteristics…”) remain consistent with the original version, since the feature-importance ranking showed similar tendencies (especially for Funding Amount and Max JIF (5 years)). Therefore, our conclusions remain valid under the bias-free sampling framework.

For further details and revised figures, please refer to the updated sections from Section 4 onward in the revised manuscript.

## Detailed Question 6

*“Line 162: “To mitigate class imbalance, we downsampled the larger group to match the smaller group’s size via random sampling.” Which groups do you exactly refer to?”*

**Response:**
Thank you for your question. The class imbalance adjustment refers to the difference in sample sizes between the Positive (growing) and Negative (non-growing) startup groups. As shown in the “Number of Verification Data / Remaining Data” row of Table 1, this adjustment was applied in Cases 1, 2, and 4. To ensure balanced training data, the larger group (Negative) was randomly downsampled to match the size of the Positive group prior to model training. The following text has been added in the revised manuscript.

- **Lines 225–226:** “This adjustment was applied in Cases 1, 2, and 4, as the number of Negative (non-growing) startups exceeded that of Positive (growing) ones, as indicated in the “Number of Verification Data / Remaining Data” row in Table 1..”

## Detailed Question 7

*“Line 249: “In summary, key characteristics of researchers contributing to the growth of university-originated startups include securing a cumulative KAKENHI grant amount of at least 10 million yen as a principal investigator, and/or being the author of papers with JIF ≥20 in the biomedical field or JIF ≥10 in the non-biomedical field.” However, what should not implied by the model anal-ysis is that it also holds the other way around. A high cumulative budget and high JIFs do not re-sults in start-ups.”*

**Response:**
Thank you for this important clarification. We fully agree that the results of our model should not be interpreted in a causal manner—that is, high research funding or publication impact does not necessarily lead to startup formation.

To clarify this point, we have explicitly stated in the revised manuscript that the feature importance and breakdown tree analyses represent statistical associations rather than causal relationships. The following sentence was added in Section 4.3:

- **Lines 283–285:** “It should be noted that the feature importance scores presented here represent statistical associations identified by the model and do not imply direct causal relationships between researcher characteristics and startup growth.”

This addition ensures that readers clearly understand the interpretive limits of our analysis and that the identified researcher characteristics are correlational indicators, not causal determinants.

## Detailed Question 8

*“Line 273: “... a representative sample …” How can this be representative, if it is very selective?”*

**Response:**
Thank you for pointing out this important issue. We agree that the dataset, while carefully constructed, cannot be considered fully representative due to the selective nature of publicly available data in Japan.
To address this, we revised the Conclusion section to clarify this limitation and avoid implying complete representativeness. The following sentences have been added

- **Lines 283–285:** “It should also be acknowledged that our analyzed sample may not fully represent the entire population of Japanese university-affiliated startups. Since publicly available information on such startups remains limited in Japan, some degree of selection bias may remain.”

This revision clarifies that our analysis is based on the most comprehensive data currently available but recognizes its inherent limitations regarding representativeness.

## Detailed Question 9

*“Line 281: “This study aimed to develop an evaluation model…”. I do not think this is an evaluation, but it is rather an identification model. Causality is not analyzed at all and is unclear. There could be selection effects in the way that only highly visible researchers are (self) selected to contribute to spin-offs. If they do perform good in the founding activity is not analyzed, so it is not a perfor-mance evaluation.”*

**Response:**
Thank you for the valuable comment. We agree with your point and have replaced the term “evaluation model” with “identification model” throughout the manuscript to avoid implying causality. We also clarified the related descriptions accordingly to reflect this revision.

## Detailed Question 10

*“Line 292: “The evaluation model and insights obtained through this study are expected to contrib-ute to the development of a more robust decision-making foundation for deep-tech investment.” I think the conclusions are a little short and too general. At least, putting them in perspective with current practices or program goals would have been appreciated.”*

**Response:**
Thank you for the helpful suggestion. We have expanded the **Conclusion** section to place our findings in the context of Japan’s current startup policies and initiatives, such as the *Startup Development Five-Year Plan* and university-based deep-tech ecosystem programs. The revised conclusion now links our study’s implications more clearly to ongoing national strategies and practical applications.
