# Supplementary material for: Identifying researcher characteristics driving growth in Japanese University-originated deep-tech startups: A machine learning approach
Source: PLoS One. 2026 Apr 3;21(4):e0346137. doi: 10.1371/journal.pone.0346137 (PMC13048777; doi:10.1371/journal.pone.0346137)
Supplement: Attachment — Submitted filename: 2nd Response_to_Reviewer#2_Mizuhara.docx [file pone.0346137.s003.docx]

# Response to Reviewer 2

We sincerely thank Reviewer #2 for the thoughtful and detailed comments. We take your general point that parts of our previous responses and manuscript text could be read as overstating what can be concluded from the present design—particularly where wording might suggest a “bias-free” sample, causal explanation, or causal effects. Our study relies on observational data and necessarily uses a subset of ventures that can be consistently linked across databases and matched to researcher identifiers; therefore, it should be framed as a selection of observations rather than a random sample. In this revision, we have revised the manuscript accordingly by (i) removing and reframing language that may imply bias-free sampling, (ii) making the selection and linkage process explicit and reproducible, and (iii) systematically replacing causal-sounding expressions with wording that reflects association and prediction. We clarify that all findings should be interpreted as model-derived associations that support predictive identification, not as evidence of causal effects; any additional regression analyses are included solely to provide interpretable association summaries and do not imply causality.

## Comment 1

*“However, I am still concerned about the data treatment and the massive filtering of the data. The authors have addressed my comments, but not to my full satisfaction. In their answers to my comment on the original paper they state: “Therefore, our conclusions remain valid under the bias-free sampling framework.” I strongly disagree that what they present in their paper is a bias-free sampling framework. This statement reflects the perspective of the authors and summarizes my still existing concerns. The authors cannot know, according to their analytical framework, if the observations they have are unbiased as they do not control for any biases. In addition, this is not a sample, but a selection of observations. Even if the selection is well grounded on the data, it is still a selection. As the selection criteria (e.g. data reliability) might be correlated with the outcome (dependent variable), it might be biased. Further treatment of the data on high and low performing researchers even increased my concerns.”*

**Response:**
Thank you for your detailed feedback. We agree that our earlier wording—especially the reference to a “bias-free sampling framework”—was not sufficiently supported. We have therefore removed/reframed this statement and now explicitly describe our dataset as a selection of observations constructed under practical constraints of data availability and cross-database linkage feasibility, rather than as a bias-free random sample.
　To address your concerns, we (i) expanded the methodological description, “3.	Data Cleaning / Setting Variables”, to make the inclusion/exclusion rules and linkage process more transparent and reproducible, and (ii) strengthened “Conclusion” to explicitly acknowledge the remaining selection concern and to caution against over-generalization to the full population.
　Regarding the point on “high/low performing researchers,” we would like to clarify that we do not perform a high–low subgroup treatment in our analysis; researcher-related explanatory variables are handled primarily as continuous measures within the modeling framework.

The concrete revisions are shown below.

- **Line 94**
  This database contains ~~detailed~~ information on university-affiliated startups, such as business descriptions,
- **Line 112**
  Figure 1 summarizes the data integration and cleaning process used to construct the final analytical sample.The “University-Originated Venture Database” contained 967 listings, which were matched with “STARTUP DB” using corporate numbers as the primary identifier. As a result, 357 startups were successfully linked between the two databases. A total of 610 entities were excluded at this stage. This reduction reflects structural differences in database definitions rather than data loss. Specifically, the University-Originated Venture Database maintained by the Ministry of Economy, Trade and Industry (METI) allows the inclusion of a broad range of organizational forms, such as general incorporated associations, and does not require listed entities to have conducted external fundraising. In contrast, STARTUP DB focuses on venture companies with identifiable corporate registration and financial activity, resulting in a narrower and more investment-oriented subset.
- **Line 540**
  It should also be acknowledged that our analyzed sample may not fully represent the entire population of Japanese university-affiliated startups, as the dataset was constructed under practical constraints of data availability and linkage feasibility across sources. Therefore, expanding, standardizing, and integrating relevant databases remain essential to strengthen the robustness and external validity of this line of research. At the same time, it is important to clarify that our analytical dataset should be regarded as a selected subset conditioned on successful cross-database linkage and the availability of researcher identifiers and related records, rather than as a bias-free random sample. These inclusion conditions could plausibly be associated with fundraising visibility and disclosure practices, and therefore the observed associations are best interpreted as patterns within this selected, investment-observable subset. Accordingly, generalization to the full population should be made with caution.

## Comment 2

*“I also think that the analytical framework could be enhanced. The authors should consider a logistic regression analysis at the end of section 4 of their paper. This would maybe clarify a lot of open issues. The classification work can then be described as it is, but it is just the pre-analysis for the regression analysis. The regression would have startup growth (Negatives/Positives; 0/1) as the dependent variable and the explanatory variables would be company characteristics (e.g. startup from universities A, B, C, …; sectors: biomedical/else) as well as characteristics of the re-searchers (excellence; experience/scientific age, …). This would then allow for the reporting of significance levels and maybe even causal effects.”*

**Response:**
We thank the reviewer for this helpful suggestion, which prompted us to clarify the interpretation of our findings. In response, we added a logistic regression analysis at the end of Section 4 (Section 4.4). Using the same binary growth labels defined in Table 1, we report odds ratios with 95% confidence intervals and p-values for a parsimonious set of explanatory variables aligned with the interpretation of the machine-learning models. We present these results to aid interpretation of the ML findings, without implying causal effects.

## Detailed Question 1

*“Line 94: “This database contains detailed information on startups”. If this is really the case, then this information can be used for a „non-response analysis“ of the 610 companies that were excluded from the analysis. What are their core characteristics?”*

*“Line 153-155: “This reduction occurred because only those startups that explicitly disclosed their researchers in the original database and could be reliably matched to financial records were re-tained to ensure data consistency and to avoid missing values in explanatory variables.” While the reason for selection might be reasonable and convincing, the question still is what the effect on the outcome of the study is. At least some information is available on these 610, so why not analyzing them?”*

**Response:**
Thank you for the comment. We agree our wording was misleading. The 610 excluded entities could not be matched to STARTUP DB (i.e., they do not exist there), so we cannot retrieve comparable firm-level variables and therefore cannot conduct a non-response analysis of their characteristics. This mismatch mainly arises because the University-Originated Venture Database uses a broader definition of “university-originated ventures” and may include entities that are not strictly startups (e.g., general incorporated associations). We apologize for the inaccurate phrasing “detailed information on startups” and have revised the manuscript by removing “detailed” to avoid overstatement.

## Detailed Question 2

*Line 115/116: “… we filtered startups by the main technology field of their core product or service as listed in the Venture Database.” Please be more precise here how you filtered. I assume this is targeting the deep-tech companies?”*

**Response:**
Thank you for the comment. We used the METI University-Originated Venture Database field “Main technology field related to the core product/service” to subset our sample. This field is recorded as one of the database categories, such as Bio/Healthcare, Medical devices, Electronics, Robotics, Materials, Environment/Energy, AI/IoT, Aerospace, Software/Apps, and Other.

In our study, the METI database is treated as a population of university-originated ventures that commercialize university research outputs, which are in essence deep-tech oriented. We therefore did not apply an additional “deep-tech vs. non-deep-tech” filter; instead, we only extracted the Bio subset by retaining firms classified as “Bio/Healthcare” in this field (and excluding the other categories). We will revise the manuscript to explicitly state these category labels and our filtering rule.

We have also added this definition and filtering rule as a below note to **Table 1**, so that readers can clearly see how the Bio subset was constructed.

- **Line 156**
  Startups were filtered using the METI University-Originated Venture Database category “main technology field related to the core product/service.” In the Bio subset, we retained only firms classified as “Bio & Healthcare” and excluded other categories

## Detailed Question 3

*“Line 113, Figure 1: The numbers you report in the text is 194 companies, which does not corre-spond to any of the four cases. The maximum is 190 so what happened to the 4 missing cases? In addition, given that some of the cases have really low numbers (102 and 66), did you conduct any outlier analyses? Outliers might have a high impact on the overall outcome.”*

**Response:**
Thank you for pointing this out. We believe “Figure 1” is a misreference; the relevant information is reported in Table 1. The number “194 companies” in the text refers to the total number of observations used in the balanced training setup, i.e., 95 × 2 (Negative/Positive) plus the remaining 4 Positive cases (“Remaining Positive = 4” in Table 1). These 4 are not “missing”; they simply indicate that after matching the number of Positive and Negative observations for machine-learning training, a small number of cases remained on the Positive side.

## Detailed Question 4

*“Line 134: “… startups founded from 2015 onward …“. Why another reduction of the number of startups and not use the pre-2015 start-ups as a control group?”*

**Response:**
Thank you for the comment. As noted in **Response to Comment 1**, the reduction was due to data reliability requirements, not selective exclusion.

## Detailed Question 5

*“Line 161/162: “Under this program, four major national universities—the University of Tokyo, Kyo-to University, Osaka University, and 162 Tohoku University—each established university-affiliated venture capital funds…” Ok, does this mean that the 194 startups selected for the analysis originated only in these four universities? It seems not as in figure 6 the top six universities are listed. However, why specifically mention these four universities here? In section 5 of the paper, it is mentioned that the top 6 universities were analyzed only. This has never been mentioned in text before section 5 (except for this figure 6). Readers are really left in the darkness concerning the selection and the observations used, which I think is not appropriate. Although some university-originated startups involve multiple researchers, each researcher is associated with only one startup to establish a consistent one-to-one relationship between startups and researchers. Please mention here how you assigned them. Furthermore, this sentence is still unclear. Are there more than one researcher per startup? This must be the case as 252 researchers are found in 194 companies. The other way around, one researcher could have been involved in more than one startup. Did you assign only one researcher to each compa-ny or did you assign one company only to each researcher? How did you aggregate the researcher data (citations, JIF, …)? Did you calculate the simple average of multiple researchers involved in one company or did you calculate a weighted average? In section 5 (see comments below) you mention that you assigned only one researcher to each of the startups by selecting the top-performer for Positives and low-performer for Negatives.”*

*And*

*Line 367: “… performance of top six universities…” This is the first time I became aware of the fact that the analyses are restricted to top 6 universities. They were listed in Figure 6, but it was never mentioned explicitly in the text. No justification is given why only these six universities have been analyzed.*

**Response:**
Thank you for these detailed comments. We agree that the manuscript required clearer explanations about (i) why four national universities were mentioned in the policy-context description, (ii) why we focused on six universities in Section 5, and (iii) how we handled cases where multiple researchers were associated with a single startup.

(1) Mention of four national universities and scope of the analytical sample.

The sentence referring to four major national universities (The University of Tokyo, Kyoto University, Osaka University, and Tohoku University) was intended only to illustrate the institutional shift and policy context surrounding the Public-Private Innovation Program and the emergence of university-affiliated VC funds. It was not meant to imply that the 194 startups in our main analytical sample originated only from these four universities. To prevent misunderstanding, we revised the relevant text in Section 3 (Data cleaning / setting variables) to explicitly state that this policy-context example does not define the university scope of the startup-level analytical sample.

- **Line 128**
  This policy context does not imply that our analytical sample was restricted to these four universities; the startups in the final dataset originated from a broader range of universities.

(2) “Top six universities” analysis and justification.

We agree that the restriction to “top six universities” should be stated more transparently. Section 5 presents a supplementary university-level assessment that applies the identified researcher characteristics to leading institutions in university-based venture creation in Japan. Specifically, we used the top six universities identified in the Survey on the Status of University-Based Ventures reported by the Ministry of Economy, Trade and Industry and Tokyo Shoko Research (23): The University of Tokyo, Keio University, Kyoto University, Osaka University, University of Tsukuba, and Tohoku University. We revised the opening of Section 5 to clarify the source and rationale for selecting these six universities and to emphasize that this university-level assessment is conceptually separate from the policy-context description in Section 3.

- **Line 497**
  This university-level assessment is intended as a supplementary application of the identified characteristics and is conceptually separate from the policy-context description in Section 3, where four major national universities were mentioned as an illustrative example of the Public-Private Innovation Program. Accordingly, the mention of those four universities does not imply that the startup-level analytical sample was restricted to any specific subset of universities.

(3) One-to-one mapping between startups and researchers; handling multiple researchers per startup.

Our dataset includes startups that can be associated with multiple researchers (194 startups and 252 involved researchers). For model training and performance evaluation, we did not aggregate researcher indicators within a startup (e.g., by simple or weighted averages). Instead, we constructed one-to-one startup–researcher datasets by repeated random sampling of one researcher per startup and summarized results across iterations, as described in the manuscript.

In addition, the rule described in Section 4.3 that selects a researcher with larger values for the Positive class and smaller values for the Negative class was introduced only for the breakdown-tree visualization, to facilitate an interpretable one-to-one mapping and illustrative decision paths. Importantly, this heuristic was not used for model training, performance evaluation, or any statistical inference. We revised the manuscript to make the scope and intent of these procedures explicit and to prevent potential misunderstanding.

- **Line 141**
  Among the 194 companies analyzed, a total of 252 involved researchers were identified, indicating that some startups are associated with more than one researcher. For model construction and evaluation, we did not aggregate researcher-level metrics within a startup (e.g., by simple or weighted averages); instead, we created a one-to-one startup–researcher dataset by selecting one researcher per startup via repeated random sampling, as described below.
- **Line 225**
  In each iteration, the sampled dataset contains at most one researcher per startup; therefore, no within-startup averaging of researcher metrics was performed
- **Line 344**
  For the breakdown-tree visualization only, when multiple researchers were associated with a single startup, we selected a single representative researcher per startup to facilitate an interpretable one-to-one mapping for the tree paths. Specifically, we chose the researcher with larger explanatory-variable values for the Positive class and the researcher with smaller values for the Negative class. This heuristic was used solely to improve interpretability of the breakdown trees and was not used for model training, performance evaluation, or any statistical inference, which relied on the repeated random sampling procedure described above

## Detailed Question 6

*“Line 191-194: “To construct a consistent one-to-one dataset for modeling, one researcher was randomly selected per startup. This random sampling was repeated five times, and the average model performance across these iterations was used to evaluate the model’s stability. This ap-proach ensured that model evaluation did not depend on any particular random draw of researchers from startups with multiple participants.” How many cases of multiple researchers did occur? If you draw a random sample of 236 researchers in 194 companies, the variation is small. Deriving a model stability and a quality check out of this is very ambitious.”*

**Response:**
Thank you for this comment. In our dataset, 36 startups were linked to multiple researchers. To construct a consistent one-to-one startup–researcher dataset for modeling, we randomly selected one researcher per startup in those cases and repeated this procedure five times. We agree that, given the limited number of multi-researcher startups, the variation introduced by this random selection is relatively small. Therefore, we have revised the manuscript to describe this procedure as a limited sensitivity/robustness check rather than a strong guarantee of model stability, and we added this clarification to the Conclusion as below.

- **Line 548**
  In addition, for startups involving multiple researchers, our one-to-one data construction relied on repeated random selection (five iterations), which should be interpreted as a limited sensitivity check rather than a definitive assessment of model stability.

## Detailed Question 7

*“Line 199: “We used 5-fold cross-validation…” the metrics used here are not validation measures, but evaluation/test measures. Given the low numbers of cases the split between learning and testing sample are already questionable, but calling these metrics cross-validation is not appropriate. In addition, these metrics are related. E.g. recall and precision constitute F1-Score…”*

**Response:**
Thank you for this comment. We agree that our wording was potentially confusing. We use 5-fold cross-validation as an evaluation procedure: in each fold, the model is trained on 4/5 of the data and evaluated on the held-out 1/5, and we report the mean performance across folds. We have revised the manuscript to consistently refer to these as evaluation metrics estimated via cross-validation, rather than “validation measures.”

We also acknowledge that several metrics are mathematically related (e.g., F1 summarizes precision and recall). To improve clarity and avoid redundancy, we revised the text in section 4 to emphasize a smaller set of complementary metrics (primarily F1 and AUC) and adjusted the wording where needed.

## Detailed Question 8

*“Line 245: “…their relative contribution…”. What does relative contribution mean? Do you mean correlation? If so, then name it that way.”*

**Response:**
Thank you for this comment. We agree that the phrase “relative contribution” was ambiguous and could be misinterpreted as referring to correlation. To clarify our intended meaning, we revised the sentence to explicitly indicate that we are referring to model-derived feature importance, i.e., the extent to which each variable influences the classifier’s decisions, rather than causal effects on startup growth.

- **Line 305**
  Therefore, we discuss these variables as factors that most influence the classifier’s decisions (feature importance), not as causal determinants of startup growth.

## Detailed Question 9

*“*Line 267: “… Funding Amount, Maximum Funding Amount, Average Funding Amount,…”. As these are just variations of the same variable, correlations cannot be interpreted as a quality indication.*”*

**Response:**
Thank you for this comment. We agree that Funding Amount, Maximum Funding Amount, and Average Funding Amount are closely related measures derived from the same underlying KAKENHI funding records. Therefore, their strong correlations should not be interpreted as an indication of “quality.” We revised the manuscript to clarify that the correlation matrix is presented to describe redundancy/overlap among related derived indicators (i.e., potential multicollinearity), rather than to infer quality.

- **Line 324**
  Because Funding Amount, Maximum Funding Amount, and Average Funding Amount are closely related measures derived from the same underlying KAKENHI funding records, the strong correlations among these variables are expected and primarily indicate redundancy/overlap among related funding indicators (i.e., potential multicollinearity), rather than any “quality” implication.

## Detailed Question 10

*“*Line 269-271: “… JCI-related variables, and h-index were more strongly correlated with each other than with quantity-based variables such as the number of papers or number of co-authors.” Also the correlations between citation-based indicators, citation rate or JIF, cannot be interpreted as a sign of quality of the analysis. They are indicators of the same conceptual category (namely research excellence) and hence multicollinear.*”*

**Response:**
Thank you for this comment. We agree that correlations among citation- and journal-based indicators (e.g., citation-based measures, citation rate, JIF-related metrics, JCI-related variables, and h-index) should not be interpreted as a sign of “quality.” These variables belong to the same conceptual category (research excellence) and are therefore expected to be highly correlated, reflecting overlap and potential multicollinearity. We revised the manuscript to clarify this interpretation in the paragraph describing Figure 4 (Correlation Matrix).

- **Line 333**
  Publication-related metrics also showed relatively high internal correlations; in particular, citation- and journal-based indicators (e.g., Max/Avg JIF (5 years), JCI-related variables, and h-index) are conceptually related measures of research excellence, so stronger correlations among them are expected and should be interpreted as overlap and potential multicollinearity rather than as evidence of “quality.” These indicators were less strongly correlated with quantity-oriented measures such as the number of papers or number of co-authors.

## Detailed Question 11

*“Line 281/282: “… Positive data, and the one with smaller values for Negative data, resulting in a one-to-one correspondence between each startup and researcher.” This comment is related to my comment above (line 165). The other reviewer had already raised this issue, but I want to re-emphasize this. I think this is a questionable approach as the good performers were taken for the good performing startups and the bad performers for the bad performing startups. The selection criteria should be the same for both as this might otherwise self-enforce the intended outcome of the study, namely bad performers perform bad and good performers good.”*

**Response:**
Thank you for reiterating this point; we appreciate your careful evaluation. We understood this concern from the previous round of review and agree that using different selection criteria for Positive and Negative classes would be problematic if it were used for statistical inference or to support causal/quantitative claims. In our study, however, this step was introduced only for the breakdown-tree visualization to create an interpretable one-to-one mapping between each startup and a single representative researcher and to illustrate typical decision paths of the classifier. Importantly, this heuristic was not used for model training, performance evaluation, or any statistical inference. The classification models were trained and evaluated using repeated random sampling of one researcher per startup, and results were summarized across iterations, as described in the manuscript. We have revised the text in Section 4.3 to make this scope and intent explicit and to avoid potential misunderstanding.

- **Line 344**
  For the breakdown-tree visualization only, when multiple researchers were associated with a single startup, we selected a single representative researcher per startup to facilitate interpretation of the tree paths. Specifically, we chose the researcher with larger values for the Positive class and the researcher with smaller values for the Negative class to create a clear one-to-one mapping for illustrative purposes. This heuristic was used solely to improve interpretability of the breakdown trees and was not used for model training or performance evaluation, which relied on repeated random sampling as described above.

## Detailed Question 13

*“Line 313: “These two variables played a central role in explaining startup growth within the model framework.” The term “explaining” implies a causal relationship, while the interpretation should be on correlations.”*

**Response:**
Thank you for this comment. We agree that the term “explaining” may be interpreted as implying a causal relationship. Since our analysis is based on model-based associations/predictive performance rather than causal inference, we revised the sentence to describe these variables as being central to predicting startup growth within the model (i.e., influencing the classifier’s decisions), not as causal explanations.

- **Line 389**
  Within the model framework, these two variables were central to predicting startup growth (i.e., to the model’s classification decisions).

## Detailed Question 14

*“*Line 344/345: “In Cases 1 and 2, where no field distinction was made, publication-quality variables exhibited less explanatory power.” This might ask for a field-specific modelling of the citation-based indicators (e.g. field-normalized citation rate).*”*

**Response:**
Thank you for the suggestion. We agree that, without field distinction (Cases 1 and 2), citation-based indicators can show reduced predictive importance due to cross-field differences in citation practices. We revised the manuscript to (i) describe this in terms of predictive importance (not “explanatory power”) and (ii) note that field-normalized indicators (e.g., field-normalized citation rates) would be preferable for mixed-field samples and are a limitation/future direction.

- **Line 471**
  This may reflect cross-field heterogeneity in citation practices, suggesting that field-normalized indicators (e.g., field-normalized citation rates) could be more appropriate when modelling publication-related metrics in mixed-field samples.
